# Sphenopalatine ganglion volumetry in episodic cluster headache: from symptom laterality to cranial autonomic symptoms

**DOI:** 10.1186/s10194-022-01534-5

**Published:** 2023-01-03

**Authors:** Jr-Wei Wu, Shu-Ting Chen, Yen-Feng Wang, Kuan-Lin Lai, Ting-Yi Chen, Shih-Pin Chen, Wei-Ta Chen, Shuu-Jiun Wang

**Affiliations:** 1 Department of Neurology, Neurological Institute, Taipei Veterans General Hospital, No. 201, Sec. 2, Shi-Pai Rd, Taipei, 11217 Taiwan; 2 Center for Quality Management, Taipei Veterans General Hospital, Taipei, Taiwan; 3 College of Medicine, National Yang Ming Chiao Tung University, Taipei, Taiwan; 4 Department of Radiology, Taipei Veterans General Hospital, Taipei, Taiwan; 5 Institute of Clinical Medicine, National Yang Ming Chiao Tung University, Taipei, Taiwan; 6 Division of Translational Research, Department of Medical Research, Taipei Veterans General Hospital, Taipei, Taiwan; 7 Brain Research Center, National Yang Ming Chiao Tung University, Taipei, Taiwan; 8 Department of Neurology, Keelung Hospital, Ministry of Health and Welfare, Keelung, Taiwan

**Keywords:** Cluster headache, Sphenopalatine ganglion, Cranial autonomic symptom

## Abstract

**Background:**

Sphenopalatine ganglion (SPG) is a peripheral structure that plays an important role in cluster headache (CH). Hence, a reliable method to measure the volume of SPG is crucial for studying the peripheral mechanism of CH. Additionally, the association between the clinical profiles and the morphology of the SPG in CH remains undetermined. This study aims to use the manual measurement of SPG volume to investigate its associations with CH, including headache laterality, cranial autonomic symptoms (CASs), presence of restlessness or agitation, and other clinical profiles.

**Methods:**

We prospectively recruited consecutive CH patients at a tertiary medical center between April 2020 and April 2022. A total of eighty side-locked, in-bout, episodic CH patients and 40 non-headache healthy controls received 1.5 T brain MRI focusing on structural neuroimaging of the SPG. The manual measurement process for SPG was under axial and sagittal FIESTA imaging, with reference T2 weight images (sagittal and axial) for localization. The inter-observer agreement of the SPG volume (both sides of the SPG from CH patients and controls) between the two observers was calculated. In CH patients, clinical profiles and the number of CASs (range 0–5) were recorded to analyze their association with SPG volume.

**Results:**

The inter-observer agreement between the two raters was excellent for the new SPG volumetry method at 0.88 (95% CI: 0.84–0.90, *p* < 0.001). The mean [SD] SPG volume was larger in CH patients than in non-headache controls (35.89 [12.94] vs. 26.13 [8.62] μL, *p* < 0.001). In CH patients, the SPG volume was larger on the pain side than on the non-pain side (38.87 [14.71] vs. 32.91 [12.70] μL, *p* < 0.001). The number of CASs was positively moderately correlated with the pain-side SPG volume (Pearson *r* = 0.320, *p* = 0.004) but not the non-pain side SPG volume (Pearson *r* = 0.207, *p* = 0.066).

**Conclusions:**

This proof-of-concept study successfully measured the SPG volume and demonstrated its associations with symptomatology in patients with episodic CH. The direct measurement of SPG provide insights into studies on peripheral mechanism of CH.

## Introduction

Cluster headache (CH) is one of the most severe pain disorders, and has a significant impact on patient quality of life, affecting 1/1000 in the general population [1]. CH is called ‘suicide headache’ because the pain is unbearable [2]. The classic presentation is strictly unilateral pain located at the orbital, supraorbital, and/or temporal regions associated with cranial autonomic symptoms (CASs) [3, 4], including lacrimation, conjunctival injection, rhinorrhea, nasal congestion, forehead and facial sweating, miosis, ptosis, and eyelid edema. The neuroanatomical substrates of the cardinal clinical presentations of CH include the central and peripheral nervous systems [3]. However, there is one unsolved issue in CH pathophysiology, that is, the underlying mechanisms for the strictly unilateral distribution of symptoms. Traditionally, neurological disorders with a strictly unilateral distribution usually point to a structural lesion or dysfunction. In CH, one study found elevated levels of calcitonin gene-related peptide and vasoactive intestinal peptide in the ipsilateral jugular vein during cluster attacks, which suggests an association between the peripheral nervous system and the secretion of neuropeptides [5].

Sphenopalatine ganglion (SPG), the largest extracranial parasympathetic ganglion of the head, may play a role in CASs in patients with CH [6–10]. In addition, the SPG is also a therapeutic target [9, 11, 12]. According to previous studies, enlargement of autonomic ganglia (i.e., cardiac and pelvic ganglia) had been observed in previous studies as an adaptive mechanism in response to increased demand for autonomic activity [13–15]. Hence, whether the repetitive activation of SPG in CH patients links to macroscopic structural changes in this ganglia warrants further investigation. MR volumetry for the peripheral nervous system has been applied to analyze the trigeminal nerve of orofacial pain disorders [16–18]. However, to the best of our knowledge, no studies have directly investigated the neuroimaging of the SPG in patients with CH as well as its clinical correlates. Hence, we hypothesized that the SPG volume is associated with the symptom laterality and number of CAS, but not all associated symptoms of CH, such as the presence of restlessness, could be linked to SPG. The aim of this study was to use the manual measurement of SPG volume to investigate the associations between the SPG volume and the clinical profiles of patients with CH, including headache laterality, the number of CASs, presence of restlessness or agitation, and other clinical profiles.

## Methods

### Study population

We prospectively recruited patients with episodic CH from the Headache Clinic of the Taipei Veterans General Hospital and non-headache healthy controls for comparisons from the community between April 2020 and April 2022. The initial diagnosis of cluster headache was made by headache specialists according to the International Classification of Headache Disorders, 3^rd^ edition (ICHD-3) [19]. We collected the clinical features of CH as well as the CASs in all recruited patients by questionnaires and chart review.

### Standard protocol approvals, registrations, and patient consents

The study protocol was approved by the Institutional Review Board of the Taipei Veterans General Hospital (2020–03-011BC), informed written consent was obtained for all participants (both CH and control groups) in accordance with the Declaration of Helsinki.

### Inclusion and exclusion criteria for CH patients and non-headache healthy controls

The inclusion criteria for CH patients were as follows: 1) patients were diagnosed by headache specialists according to the ICHD-3 diagnostic criteria of episodic CH (3.1.1), 2) age between 20 and 65 years, and 3) patients were able to undergo magnetic resonance imaging (MRI) examinations without contraindications.

**Fig. 1:**
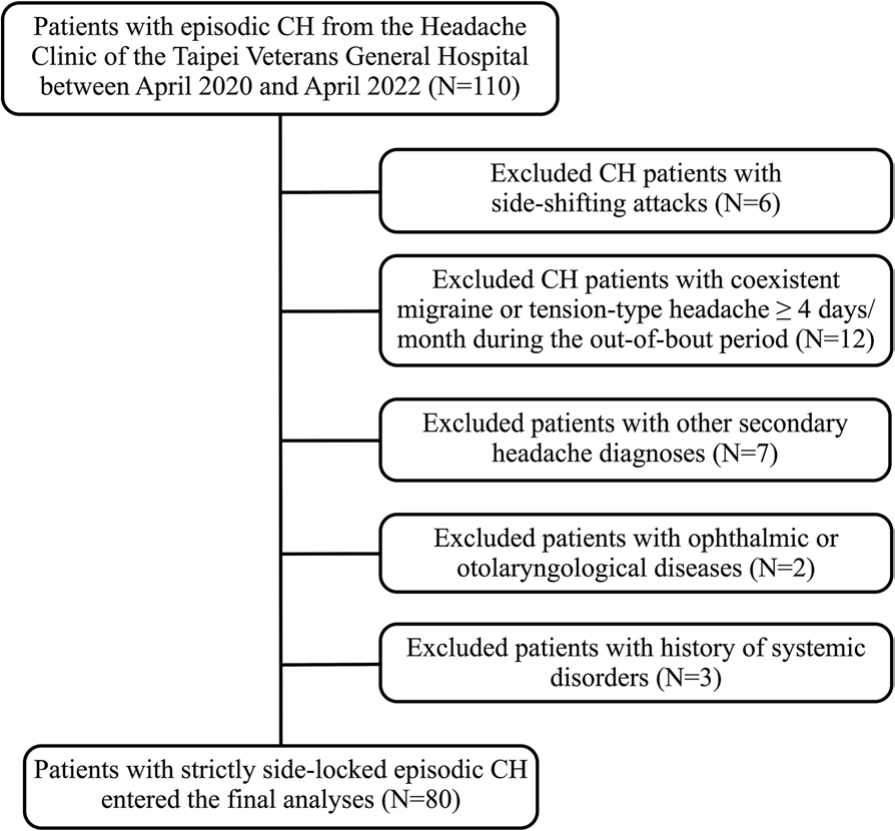
Study schematic flow chart

The exclusion criteria for the CH group included the following: 1) CH patients with side-shifting attacks; 2) CH patients with coexistent migraine or tension-type headache ≥ 4 days per month during the out-of-bout period; 3) patients with other secondary headache disorders according to the ICHD-3; 4) Patients with ophthalmic or otolaryngological diseases, 5) patients with history of systemic disorders, including uncontrolled hypertension, diabetes, chronic kidney disease, autoimmune disease, cirrhosis, and malignancy; 6) patients with a history of neurological disorders that may alter the brain structure, such as stroke and neurodegenerative disorders; 7) patients with a history of head or neck malignancies or infection that may alter the paranasal sinus structure; and 8) pregnant or lactating patients (Fig. 1).

### Control group

Non-headache healthy controls, matched by age and sex, were recruited from the nearby neighborhood or university if they did not have the following: 1) primary or secondary headache diagnoses according to the ICHD-3, 2) ≥ 1 headache episode per month, 3) a history of moderate or severe headaches, and 4–7) any of the above exclusion criteria for patients with CH. All control subjects were interviewed by a neurologist (JWW) to ensure that they did not have other headache disorders.

### Cranial autonomic symptoms

In this study, the total number of CASs (ranging from 0 to 5) was calculated based on the ICHD-3 criteria for CH, including 1) lacrimation/conjunctival injection, 2) rhinorrhea/nasal congestion, 3) forehead and facial sweating, 4) miosis and/or ptosis, and 5) eyelid edema [19].

### Specialized MR protocol for the SPG

All participants of this study received MR scans on a 1.5 T scanner (Optima™ MR450w GEM, GE Healthcare, United States). Technical parameters for MR scans were as follows: 1) Two-dimensional sagittal T2-weighted images: repetition time (TR)/echo time (TE) = 3138/102 ms, slice thickness = 2 mm, matrix: 320 × 224, field of view (FOV) = 16 cm × 16 cm, number of excitations (NEX) = 6, voxel size 0.5 × 0.714x2 mm. The scanning range of the sagittal T2-weighted images was about 24 mm, focusing on the pterygopalatine fossa, which covered bilateral SPG and vicinity. 2) Two-dimensional axial T2-weighted images: TR/TE = 3973/103 ms, slice thickness = 2 mm, matrix: 320 × 224, FOV = 16 cm × 16 cm, NEX = 6, voxel size 0.5 × 0.714x2 mm. Axial T2-weighted images also focused on the SPG, and the scanning range was about 36 mm. The upper border was the lower margin of the lens, and the lower border was the line tangential to the upper two-thirds of the maxillary sinus [17, 20–22]. 3) Two-dimensional axial T1-weighted images: TR/TE = 9.6/3.8 ms, slice thickness = 1 mm, matrix: 288 × 224, FOV = 24 cm × 18 cm, NEX = 1, voxel size 0.83 × 0.8x1 mm. The axial T1-weighted images was performed on the whole brain. 4) Three-dimensional axial and sagittal fast imaging employing steady-state acquisition (FIESTA): TR/TE = 6.5/2.0 ms, slice thickness = 0.8 mm, matrix: 352 × 352, FOV = 22 cm × 22 cm, NEX = 1, voxel size 0.625 × 0.625x0.8 mm. The sagittal FIESTA imaging was performed on the whole brain. All sequence were performed without slice gap [17, 20–22]. The total acquisition time of the whole specialized protocol for SPG was 25 min 46 secs.

Both patients and healthy controls received the same protocols, and CH patients underwent head MRI during cluster bouts. To ensure the quality of the MRI images, we performed all MRI studies (CH and control) on the same scanner. We performed routine quality control twice per week to ensure the imaging quality. Moreover, all scans of this study were operated by the same specialized radiologic technologist (TYC). During MRI scanning, our research team, including a radiologic technologist (TYC) and neuroradiologist (STC), were on site to check the imaging quality immediately after the scanning. When the imaging was not qualified, we repeated the scan until the imaging fulfilled the requirements in order to avoid motion artifacts and maintain the signal-to-noise ratio.

### Measurement

**Fig. 2:**
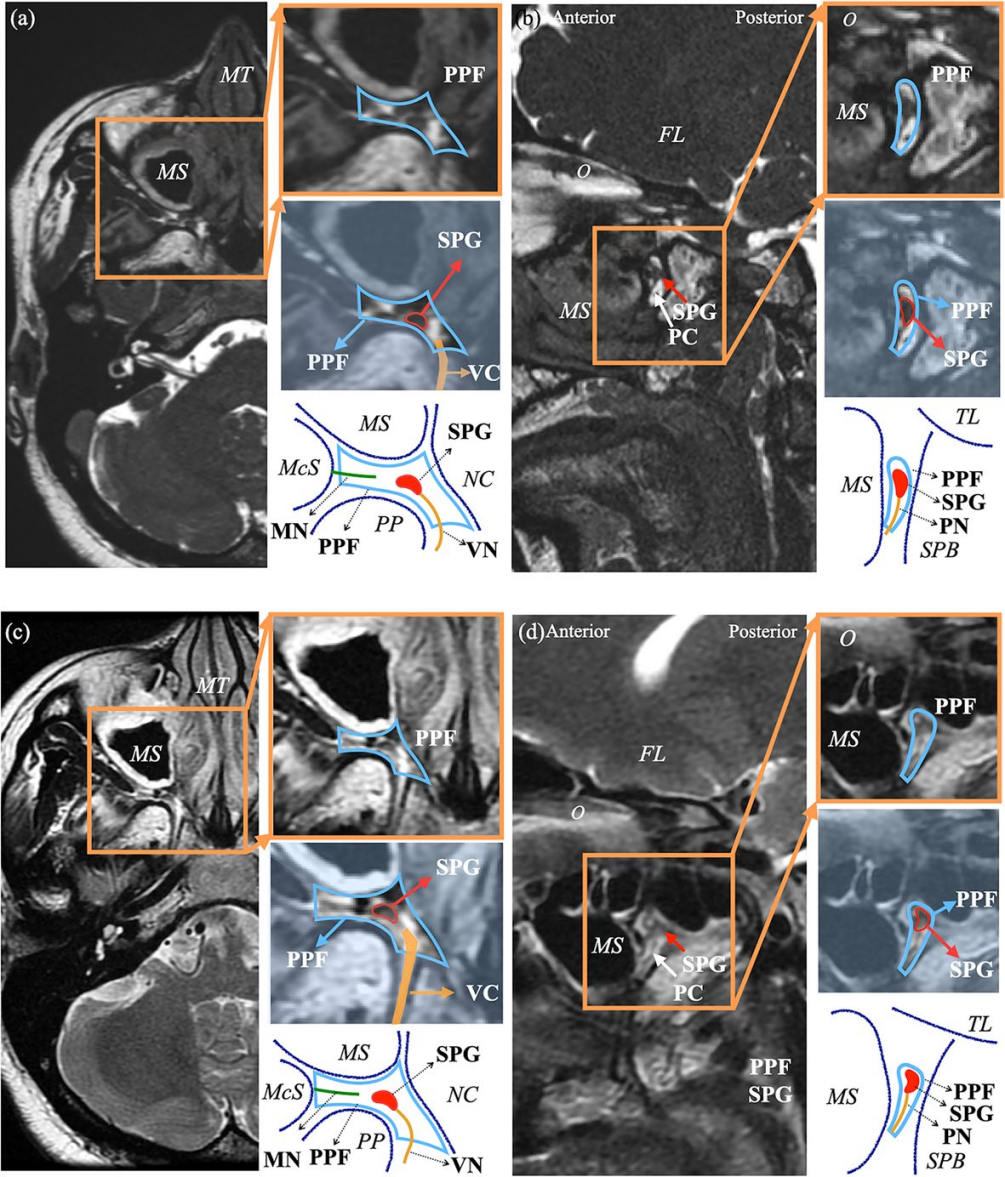
Visualization and measurement of the SPG. Axial (**a**) and sagittal (**b**) FIESTA MRI, axial (**c**) and sagittal (**d**) T2-weighted MRI depicting the sphenopalatine ganglion (SPG) (the red arrow is positioned in the center of the SPG, blue arrow is positioned at the pterygopalatine fossa [PPF]). The SPG has a crescent shape and intermediate signal intensity. The image surrounded by the orange rectangle is the ‘zoomed-in’ image. The light blue polygon depicts the border of the PPF. The oval red line is the border of the SPG. The dark blue line depicts the adjacent structure of PPF. FL cerebral frontal lobe, McS masticator space, MN maxillary nerve, MS maxillary sinus, MT middle nasal turbinate, NC nasal cavity, O orbit, PC palatine canal, PN palatine nerve, PPF pterygopalatine fossa, PP pterygoid process, SPB sphenoid bone, TL temporal lobe, VC vidian canal, VN vidian nerve

The SPG volume was manually measured by two neuroimaging specialists (STC and JWW) blindly and independently by using axial and sagittal FIESTA imaging (SPG diameters: axial and sagittal FIESTA; SPG volumetric: sagittal FIESTA), with the reference of T2-weighted images (axial and sagittal) for localization (Fig. 2). Of note, our picture archiving and communication system (PACS) allows labeling and measuring the same structure synchronously in different neuroimaging sequences. The first step of SPG volumetry was calculating the cross-sectional area of SPG on each image slice by delineating a freehand region of interest around the ganglion via institutional radiology software (SmartPACS, Taiwan Electronic Data Processing Corp.). Then, the whole SPG volume was subsequently calculated as the sum of the cross-sectional area multiplied by the slice thickness. The inter-observer agreement of the SPG volume (both sides of the SPG from CH patients and controls) between the two observers (STC and JWW) was calculated to ensure that the method was reliable. The results of the measurement by the neuroradiologist (STC) with 7 years of experience who specialized in extracranial structures of the head and neck system were used for final analyses. In this study, we also recorded the shape of SPG based on the in vivo morphological classification proposed by Bratbak et al., including round, elongated, and crescent shapes [20].

### Statistics

SPSS version 22.0 (SPSS Inc., Chicago, Illinois, USA) was used in the data analyses. Each rater measured the volume of SPG twice, and we calculated the intra-observer agreements in each rater and the inter-observer agreements between the two raters. For intra-observer arrangements, the intraclass correlation coefficients (ICCs) were computed to assess the agreement in the volumetric measurement of each specialist by using the two-way mixed-effects model estimating absolute agreement. For inter-observer agreement, the ICCs were computed to assess the agreement in the volumetric measurement between two neuroimaging specialists by using the two-way mixed-effects model estimating absolute agreement. According to the magnitude of these parameters, the interpretation was as follows: < 0.20, unacceptable; 0.20–0.40, questionable; 0.41–0.60, good; 0.61–0.80, very good; and 0.81–1.00, excellent [23].

The demographics and clinical profiles of CH patients were expressed as the means and standard deviations (SD) and analyzed by using t tests and chi-square tests as appropriate. The differences in the average bilateral SPG volume and diameter between CH patients and healthy controls were assessed with independent sample t-test, with a significance threshold of *p* < 0.05. In CH patients, the differences in the SPG volume between the pain and non-pain sides in the same subjects were assessed with paired t-test with a significance threshold of *p* < 0.05. The associations between the SPG volume (pain side and non-pain side) and the clinical profiles of patients, including CH duration, attack frequency, and attack duration, were exploratory analyses, which were assessed by Pearson correlations with a significance threshold of *p* < 0.05. The associations between the SPG volume (pain side and non-pain side) and presence of restlessness or agitation were also exploratory analyses, which were assessed by independent sample t-test with a significance threshold of *p* < 0.05.

## Results

### Patients

Between April 2020 and April 2022, a total of 110 patients with episodic CH were invited to join the study at Taipei Veterans General Hospital. After exclusion, a final sample of 80 patients finished the study (65 males and 15 females, right: left = 46:34) (Fig. 1). The mean (SD) age of our patients was 39.50 (9.76) years (range 21–63 years), and the mean (SD) disease duration was 15.59 (10.14) years. Regarding CH attacks, the mean attack duration was 2.28 (2.87) hours, the mean attack frequency per day was 2.01 (1.80), and the mean number of CASs during CH attacks was 3.48 (1.13). Additionally, 40 non-headache healthy controls (32 males and 8 females; mean [SD] age, 36.15 [11.57] years, range 21–63 years) were recruited for comparisons, and there were no differences in the demographics between CH patients and controls (Table 1)**.**

**Table 1:** Demographics and clinical profiles of CH patients and controls

**Demographics**	**CH patients** **(*n* = 80)**	**Non-headache healthy** **controls (*n* = 40)**	*p* value*
**Females (n, %)**	15 (18.8)	8 (20)	0.870
**Age, mean (SD), years of age**	39.50 (9.76)	36.15 (11.57)	0.121
**Smokers (n, %)**	26/80 (32.5)	13/40 (32.5)	1
**Headache laterality (right/left)**	46/34	N/A	N/A
**Duration of disease, mean (SD), years**	15.59 (10.14)	N/A	N/A
**Attack duration, mean (SD), hour**	2.28 (2.87)	N/A	N/A
**Attacks per day in bout, mean (SD)**	2.01 (1.80)	N/A	N/A
**Number of CASs (range 0–5), mean (SD)**	3.48 (1.13)	N/A	N/A
**Presence of restlessness or agitation**	54 (67.5%)	N/A	N/A

^*^Differences between continuous variables were analyzed by t-test, and the differences between categorical variables were analyzed by the chi-square test; *p* < 0.05 was considered statistically significant

### Inter-observer and intra-observer agreement for the measurement of the SPG volume

The intra-observer agreement of STC and JWW was high, with intraclass correlation coefficients (ICCs) of 0.87 (95% CI: 0.84–0.90, *p* < 0.001) and 0.82 (95% CI: 0.78–0.86, *p* < 0.001), respectively. The inter-observer agreement between two observers (STC and JWW) for a total of 240 SPG (both sides of the SPG from 80 CH patients and 40 controls) was excellent, with an ICC of 0.88 (95% CI: 0.84–0.90, *p* < 0.001). The results of the measurement by the neuroradiologist (STC) who specialized in extracranial structures of the head and neck system were used for final analyses.

### SPG volume and shapes: CH patients vs. controls and headache laterality

**Fig. 3:**
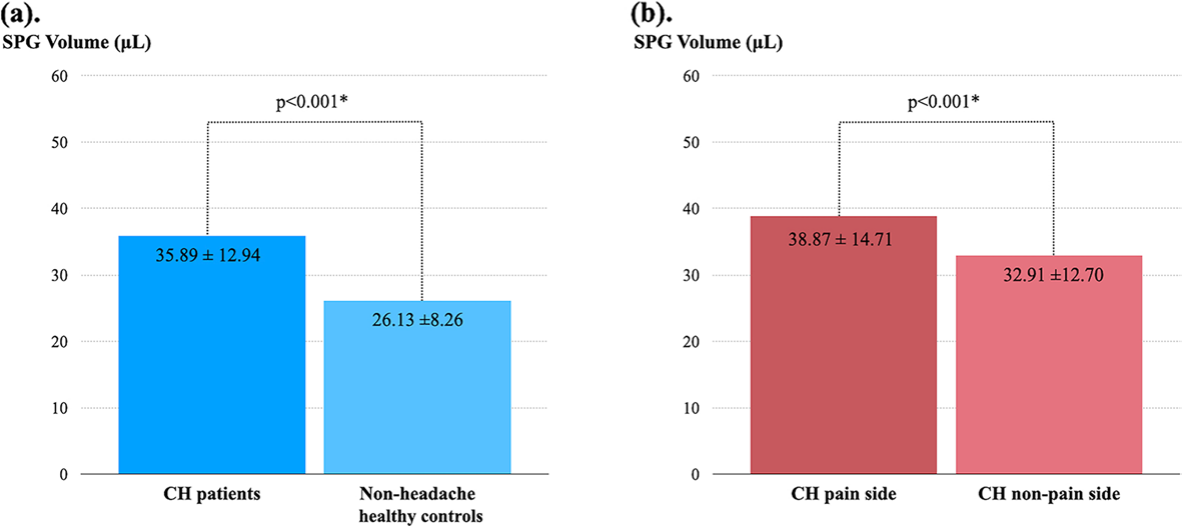
**(a)** The SPG volume of CH and control group (**b**) The SPG volume of the pain side and non-pain side

The mean [SD] bilateral average SPG volume was larger in CH patients (35.89 [12.94] μL) than controls (26.13 [8.62] μL, *p* < 0.001). Additionally, the mean [SD] width and height of the SPG were larger in CH patients than controls (width, CH vs. controls: 4.11 [0.66] vs. 3.68 [0.49] mm, *p* < 0.001; height, CH vs. controls: 4.54 [0.89] vs. 4.13 [0.92] mm, *p* = 0.020). Regarding the headache laterality of CH patients, the SPG volume (mean [SD]) was larger on the pain side than on the non-pain side (pain side vs. non-pain side: 38.87 [14.71] vs. 32.91 [12.70] μL, *p* < 0.001). The measurements of the SPG in each group are shown in Table 2 and Fig. 3. However, there was no difference in the distribution of each shape of SPG between CH patients and controls, as well as pain-side and non-pain side (Table 3).

**Table 2:** Comparisons of SPG volume and diameters (CH patients vs. non-headache healthy controls and pain side vs. non-pain side)

	**SPG volume and diameters**	**CH** **(*n* = 80)**	**Controls** **(*n* = 40)**	***p* value** **(CH vs. controls)**^a^	**CH patients**		***p* value** **(pain vs. non-pain side)**^b^
		Mean of bilateral SPG	Mean of bilateral SPG		Pain side (*n* = 80)	Non-pain side (*n* = 80)	
**Volumetry**	**Volume, mean (SD), µL**	35.89 (12.94)	26.13 (8.62)	< 0.001#	38.87 (14.71)	32.91 (12.70)	< 0.001#
**Diameter measurements**	**Depth, mean (SD), mm**	2.09 (0.4)	2.09 (0.50)	0.955	2.14 (0.49)	2.04 (0.47)	0.118
	**Width, mean (SD), mm**	4.11 (0.66)	3.68 (0.49)	< 0.001#	4.24 (0.81)	3.99 (0.86)	0.029#
	**Height, mean (SD), mm**	4.54 (0.89)	4.13 (0.92)	0.020#	4.48 (1.13)	4.61 (1.0)	0.355

^a^Differences in volume and diameters between CH and non-headache healthy controls were analyzed by independent samples t-test

^b^Differences in volume and diameters between the pain side and non-pain side were analyzed by paired t-test

^#^*p* < 0.05 was considered statistically significant

**Table 3:** Comparisons of SPG shapes (CH patients vs. non-headache healthy controls and pain side vs. non-pain side)

	**SPG volume and diameters**	**CH** **(*n* = 80)**	**Controls** **(*n* = 40)**	***p* value** **(CH vs. controls)**^a^	**CH patients** **(*n* = 80)**		***p* value** **(pain vs. non-pain side)**^b^
		160 SPG from both sides	80 SPG from both sides		Pain side (80 SPG)	Non-pain side (80 SPG)	
**Shape**	**Round (Number of SPG,%)**	87 (54.4)	41 (51.3)	0.723	40 (50.0)	47 (58.8)	0.531
	**Crescent (Number of SPG,%)**	56 (34.0)	32 (40.0)		31 (38.8)	25 (31.2)	
	**Elongated (Number of SPG,%)**	17 (10.6)	7 (8.7)		9 (11.3)	8 (10.0)	

^a^Differences in shape of bilateral SPG between CH and non-headache healthy controls were analyzed by chi-square test, *p* < 0.05 was considered statistically significant

^b^Differences in shape of SPG between pain-side and non-pain side of CH patients were analyzed by chi-square test, *p* < 0.05 was considered statistically significant

### Phenotypes of CH and the SPG volume

#### CASs and sense of restlessness or agitation

In this study, only one (1.3%) CH patient did not have any CASs, and 54 (67.5%) CH patients had sense of restlessness or agitation. Regarding the association between the SPG volume and the CASs of CH patients, the total number of CASs was positively moderately correlated with the pain side SPG volume (Pearson *r* = 0.320, *p* = 0.004) but not the non-pain side volume (Pearson *r* = 0.207, *p* = 0.066). On the other hand, presence of restlessness or agitation did not associate with the either pain side (presence vs. absence: 39.86 [15.37] vs. 36.80 [13.27] μL, *p* = 0.386) or non-pain side SPG volume (presence vs. absence: 33.66 [13.73] vs. 31.35 [10.27] μL, *p* = 0.450).

#### Disease duration, attack duration and the frequency of attacks

There were no associations between the pain side or non-pain side SPG volumes and the duration of disease (pain side: Pearson *r* = 0.001, *p* = 0.996; non-pain side: Pearson *r* = 0.007, *p* = 0.950), attack duration (pain side: Pearson *r* = 0.027, *p* = 0.816; non-pain side: Pearson *r* = -0.005, *p* = 0.963), or attack frequency (pain side: Pearson *r* = 0.063, *p* = 0.593; non-pain side: Pearson *r* = 0.137, *p* = 0.241).

## Discussion

Using the manual MR volumetry for SPG, we first demonstrated that the SPG volume was not only larger in patients with CH than in controls but also larger on the pain side than the non-pain side, but there were no differences in the shape of SPG between CH patients and controls, as well as pain-side and non-pain side. Moreover, we found a higher the number of CASs associated with a larger SPG volume.

Prior studies proposed the importance of SPG in the pathophysiology of CH, mainly focusing on the CASs during CH attacks [24–27]. However, there is a paucity of studies to date that have investigated the association between symptomatology and the volume of the SPG, which is a primary focus of the present study. Hence, the strength of the present study is that by using a specialized protocol for the visualization and measurement of the SPG, we showed a high inter-observer agreement (ICC = 0.88). Regarding the external validity, the SPG diameters measured by our specialized protocol were generally similar to those in a cadaver study (mean diameter: 3.58 mm) [28]. In addition, one MRI study by Bratback et al. analyzed the SPG diameters in a mixed cohort of 10 patients with CH and 10 patients with chronic migraine, and their diameters were close to those of the present study (Bratback et al.’s study: mean [SD], depth 2.1 [0.5] mm, width 4.2 [1.1] mm, height 5.1 [1.4] mm; present study: depth 2.09 [0.50] mm, width 4.11 [0.66] mm, height 4.54 [0.89] mm from 80 CH patients) [20]. However, caution should be taken that their study cohort was not purely CH patients, and their study did not include a healthy control group [20]. Therefore, direct comparisons of the SPG diameters of these studies are not likely.

Our study demonstrated that the SPG volume was associated with headache laterality and CASs during CH attacks. Currently, little information is available about the association between SPG volume and CH symptomatology due to the difficulty in vivo measuring the size of SPG. However, enlargement of autonomic ganglia had been observed in the cardiac and pelvic ganglia [13–15]. In the heart, hypertrophy of the autonomic ganglia, hyperinnervation, and neuronal remodeling is triggered by certain pathological stress conditions, such as ischemia [13]. On the other hand, pelvic ganglion hypertrophy had been reported in animal models that received contralateral ganglionectomy, which might be a physiological compensatory response to increased demand for autonomic activity [15]. Hence, the enlarged ganglia, such as larger symptomatic side SPG in CH, might be an adaptive mechanism to maintain normal physiological function [13, 29]. However, our research findings should not be interpreted as SPG being the sole neuroanatomical substrate for the symptoms of CH attacks. For example, the circadian and circannual features of CH attacks are better explained by the involvement of the suprachiasmatic nuclei of the anterior hypothalamus [3, 30, 31]. Currently, hormonal, genetic, neuroimaging studies suggest that the hypothalamus may be the generator of CH attacks [32]. and the paraventricular hypothalamic nucleus has direct projections to the superior salivatory nucleus (SSN) [3, 33]. The SSN forms a reciprocal connection with the SPG, called the trigeminal-autonomic reflex, which is associated with increased cranial parasympathetic activity during CH attacks [34]. Based on these findings, the enlargement or “hypertrophy” of the SPG in patients with more CASs might be due to the hyperfunction of parasympathetic activities after the repetitive generation of CH attacks.

The present study has several implications. First, this is one of the pioneering studies that developed a method for the quantitative measurement of the SPG and, moreover, disclosed its association with headache laterality and CASs. It would be interesting to test whether SPG volumetry might be useful in the future to diagnose patients with episodic CH or the development of a neuroimaging-based headache classification system. Second, this volumetry might be used to study disorders manifesting with CASs or other trigeminal autonomic cephalalgias. However, the study also has limitations that must be considered when interpreting the results. First, our study results could not be extrapolated to chronic CH because we only recruited patients with episodic CH. Because of the rarity of chronic CH in Asians [35], we, for homogeneity, did not include patients with chronic CH. Second, since this is a proof-of-concept study and we hypothesized the SPG volume associated with symptomatology during CH attacks, we only analyzed the SPG volume among in-bout CH patients. Based on the research findings, it is reasonable to hypothesize that the SPG volume might differ between in and out-of-bout. Thus, comparing the SPG volume between in and out-of-bout CH could be a future direction of study. Third, the proportion of CH without CASs was about 3% in Taiwanese and Italian studies, and only one patient in our cohort did not experience any CASs during CH attacks [36, 37]. Hence, we cannot compare the SPG volume in CH patients with or without CASs in the present study. Fourth, the manual measurement of SPG is an intrinsic limitation. To minimize the possibility of manual measurement errors, the measurements were performed by two raters blinded to the patients' clinical profiles, and good intra-observer and inter-observer reliability was observed. Fifth, our CH patients came from one tertiary medical center, which may represent a relatively more severe CH population. Therefore, our results should be generalized to other samples or populations with caution. Last but not least, many patients’ CASs were not witnessed during their CH attacks; therefore, several CASs may not be correctly reported. On the other hand, our CAS data were collected by a specialized questionnaire, which ensured the reliability of the number of CASs in the present study.

## Conclusions

CH patients had larger SPG volume than non-headache controls. In side-locked episodic CH patients, the SPG volume was larger on the pain side than on the non-pain side. Additionally, the number of CASs was positively correlated with the SPG volume. The direct measurement of SPG provides insights into future studies on the peripheral mechanism of CH.

## Data Availability

Data would be available on reasonable request.
